# Chromosomal integration of an avian oncogenic herpesvirus reveals telomeric preferences and evidence for lymphoma clonality

**DOI:** 10.1186/2042-4280-1-5

**Published:** 2010-12-07

**Authors:** Charmaine M Robinson, Henry D Hunt, Hans H Cheng, Mary E Delany

**Affiliations:** 1 Department of Animal Science, University of California, Davis, CA 95616, USA; 2 USDA-ARS Avian Disease and Oncology Laboratory, East Lansing, MI 48823, USA

## Abstract

**Background:**

Herpesviruses are a major health concern for numerous organisms, including humans, causing both acute and chronic infections recurrent over an individual's lifespan. Marek's disease virus (MDV) is a highly contagious herpesvirus which causes a neoplastic condition in chicken populations. Several vertebrate-infecting herpesviruses have been shown to exist in an integrated state during latent periods of infection. However the status of MDV during latency has been a topic of debate.

**Results:**

Here we employed high-resolution multi-color fluorescence in situ hybridization (FISH) to show integration of MDV at the telomeres of chicken chromosomes. Cytogenomic mapping of the chromosomal integrations allowed us to examine the clonal relationships among lymphomas within individuals, whereas analysis of tumors from multiple individuals indicated the potential for chromosomal preferences.

**Conclusions:**

Our data highlight that substantive genome-level interactions between the virus and host exist, and merit consideration for their potential impact and role in key aspects of herpesvirus pathobiology including infection, latency, cellular transformation, latency-breaks and viral evolution.

## Background

Herpesviruses are a large group of DNA viruses that cause disease in many organisms [1,2]. The ability of herpesviruses to occupy diverse biological niches, the wide range of cell types targeted for primary infection and latency, along with acute and chronic infection symptoms, contribute to the significance and relevance of herpesvirus research. In particular, human herpesviruses are a major public health concern due to the prevalence of such viruses within and among populations worldwide and the associated pathologies. Two of the eight disease-causing human herpesviruses, Epstein-Barr virus (EBV) and Kaposi's sarcoma virus (KSHV), are associated with neoplastic transformation. A closely related avian herpesvirus, Marek's disease virus (MDV; family *Herpesviridae*, genus *Mardivirus*, species *Gallid herpesvirus 2*) also exhibits oncogenic properties inducing lymphoid tumors in chickens.

MDV rapidly infects B cells and subsequently transforms T cells during pathogenesis of the highly contagious neoplastic disease of chickens known as Marek's disease (MD). Since discovery [3], MD has been extensively studied and utilized as a valuable biomedical model for human herpesvirus infection and viral induced malignancies [4,5]. MDV is of great concern in agriculture, causing large economic losses as well as impacting animal well-being. Despite current control through the use of vaccines, viral evolution leads to increasing virulence and vaccine breaks on a cyclical basis [6,7].

A fascinating biological property that MDV shares with all herpesviruses is the ability to persist indefinitely in the host and evade immune response by establishing a latent infection. The molecular mechanisms controlling lytic replication and latent persistence of herpesviruses have been the subject of intense study in recent years, aided largely by advances made in the field of viral genetics. Although new information has emerged from these studies regarding mechanisms of the lytic cycle, factors controlling latency and tumorigenesis remain poorly understood. MDV presents a unique model system in which to explore the mechanisms of herpesvirus latency and virus-induced tumor formation.

Another intriguing aspect of herpesvirus infection is that both episomal and linear-integrated forms of the virus are present in the host [8]. One consideration is that latency, and potential reactivation of the virus, could be achieved in part through genomic interactions between the host and virus, evasion through integration and recurring infection through emergence/release. Evidence for herpesvirus integration into human chromosomes was reported for Human Herpesvirus 6 (HHV-6) and EBV [9-14]. Since Delecluse and Hammerschmidt (1993) first reported on the potential for MDV to exist in an integrated state, it has been a subject of debate as to whether the virus integrates stably (and where) into the chicken genome or is merely associated and how such associations might be germane to pathogenesis and oncogenesis. Since that early report, complete genome sequences have been reported for several MDV strains and BAC vectors containing the full-length MDV genome have been cloned [15-20]. The sequencing provided data supportive of the hypothesis that MDV interacts with the chicken genome, as it was discovered that the circular viral genome encodes several orthologous host genes including the telomerase RNA (vTR) gene as well as telomeric repeats (TTAGGG) [17,18,21].

To resolve whether and where MDV integrates in the host chicken genome we employed high-resolution multi-color fluorescence in situ hybridization (FISH) using the tools developed from both the MDV and chicken sequencing efforts. We investigated the details of integration on both intra-and inter-chromosomal levels by mapping the location of MDV integrations. In depth analysis of MDV integration sites allowed us to investigate several specific objectives, including the clonal composition of MDV tumors and integration site preference of the virus. We show here that MDV specifically integrates into the telomeric regions of chicken chromosomes in MDV-induced clonal lymphomas.

## Methods

### Bird and tumor samples

Two common infection strategies were used to produce tumors. In the first case, progeny from a cross between two genetically susceptible inbred lines (USDA-ADOL 15I_5 _X 7_1_,) [22] were challenged with 1,000 plaque forming units of MDV (GA strain). The resulting 17 tumors from nine birds were processed for chromosome harvest by standard procedures. In the second strategy, peripheral blood lymphocytes harvested from MDV-infected birds were used to infect (rather than purified virus). Nine tumors from four birds were processed for chromosome harvest. Animal experimental procedures, care and management were approved by the USDA-ARS Avian Disease and Oncology Laboratory Animal Care and Usage Committee.

### Fluorescence in situ hybridization (FISH)

The FISH procedures were standard [23,24] except that a telomere oligonucleotide (10 μM (5' TTAGGG 3')_7_,) was included in the hybridization to provide competitor DNA to ensure blockage of MDV telomeric repeats [21] from hybridizing to chicken telomeric repeats (see also control experiments described in the results). To allow for multiple hybridizations, slides were cleaned and re-hybridized [25].

### Probes and labeling

Table 1 describes the BACs used to identify specific chicken chromosomes including GGA 6-15, 17-28, W and one linkage group (LG E22/E50C23) [24,26] (http://bacpac.chori.org; UCSC, http://genome.ucsc.edu/). For GGA 16, an rDNA probe covering the external transcribed sequence (ETS) of the 18S-5.8S-28 S repeat was utilized [27]. Probes for GGA 1-5 and the Z were not needed because these chromosomes are easily identified by their morphological features. The MDV BAC, Md11gDc1.2 was used to query MDV integration in the chicken genome by multi-color FISH (MDV of one color, chromosome probes of another, see below) [18]. A telomere-peptide nucleic acid (PNA) fluorescein probe (Applied Biosystems) was used to identify telomeric sequence repeats. Approximately 300 ng of ETS and 1 μg of each BAC clone was labeled either with digoxigenin by the DIG Nick Translation Mix (Roche, Indianapolis, IN, USA), or Spectrum Red dUTP, or Spectrum Orange dUTP by the Nick Translation Kit (Abbott Molecular, Inc., Des Plaines, IL, USA), or Cy3 dUTP (Amersham Biosciences) by the Nick Translation System (Invitrogen). Probes labeled with digoxigenin were detected by rhodamine or fluorescein (FITC) anti-DIG antibody incubation (0.70 μg/slide, Roche Applied Science, Indianapolis, IN, USA).

**Table 1:** Characteristics of probes used in chicken chromosome mapping of MDV integrations.

**Chromosome**^¶^	Clone Identity	Insert Size (Kb)	Features (NCBI Accession)	Marker Position (Mb)*	References
6	CH261-169D14	226^†^	SCD (AJ297918/X60465)	18.5	[50]
7	CH261-95H15	158^†^	SP5 (NM_001044684)	19.7	
8	CH261-84K8	235^†^	ZNF326 (NM_001006533)	15.6	
9	TAM31-29A21	-	TR (AY312571)	21.5	[37]
10	TAM33-42N22	179^‡^	NEO1 (U07644)	1.30	
11	TAM32-22B17	181^‡^	ADL210 (G01630)	12.8	
12	TAM32-43M12	118^‡^	MCW198 (G31980)	12.7	
13	TAM32-5E13	173^‡^	MCW322 (G32078)	16.3	
14	TAM32-2I7	225 ^‡^	ADL200 (G01621)	39.8	
15	TAM32-87J1	-	MCW226 (G31998)	2.4	
16	ETS rDNA	3	NOR	q arm	[26,51]
17	TAM31-22I10	-	GSN (AF042795)	9.1	
18	TAM32-14L7	78^‡^	SFRS2 (X62446)	4.2	
19	TAM32-21A11	164^‡^	ACACA (X77240)	8.5	
20	TAM32-13H1	-	BMP7 (AF223970)	11.6	
21	TAM32-12A6	-	TUS0022 (AI979776)	2.5	
22	TAM32-13C2	202^‡^	CRMP62 (U17277)	0.5	
23	TAM32-27O5	-	ADL299 (G01708)	17.8	
24	TAM32-54P15	219^‡^	APOA1 (M96012)	5.2	
25	TAM32-49A10	149^‡^	LEI0345 (AJ240692)	1.7	
26	TAM32-40J16	152^‡^	MCW209 (G31986)	2.1	
27	TAM32-76B6	169^‡^	MCW233 (G31915)	1.9	
28	TAM32-4G3	167^‡^	ADL299 (G01751)	4.3	
W	TAM32-55E18	-	CW01 (D85614)	q arm	[24,52]
LG E50C23	TAM32-2A12	-	LEI0336 (AJ240683)	N/A	
	MDV: Md11gDc1.2	178^§^	N/A	N/A	[18]
	MDV: P89	43	N/A	N/A	[29]
	MDV: SN16	42	N/A	N/A	[29]

CH = Children's Hospital Oakland Research Institute, CH261 = EcoRI BAC library.

TAM = Texas A&M University, TAM31 = BamHI, TAM32 = EcoRI, TAM33 = HindIII BAC libraries [26,31].

ETS = external transcribed spacer of the 18S-5.8S-28 S rRNA gene repeat (rDNA).

BAC locations and features were obtained from US Poultry Genome Project 'Database of BACs Assigned to Chicken Genes and Markers' (http://poultry.mph.msu.edu/resources/resources.htm, May 2006 version) and/or UCSC Genome Browser (http://genome.ucsc.edu, Chicken May 2006 assembly).

Features = Genes/Markers and Genbank accession numbers (in parentheses).

**^¶ ^**Probes for GGA 1-5 and the Z were not needed because these chromosomes are easily identified by their morphological features

*Position refers to the start position of the BAC or gene/marker on the chromosome in the May 2006 chicken assembly (UCSC Genome Browser). In two cases the chromosomal coordinates are unknown, but the chromosome arm location is indicated.

^†^Obtained from the UCSC Genome Browser.

^‡^Insert size was estimated using the UCSC Genome Browser and Chicken FPC http://www.bioinformatics.nl/gbrowse/cgi-bin/gbrowse/ChickFPC as described in O'Hare and Delany 2009.

^§^Based on the Md5 strain (GenBank accession no. AF243438) [17].

"-" = unknown

N/A = not applicable

### Chromosome analysis

Chromosome and cell images were analyzed using Applied Imaging CytoVision software (3.92 GENUS). The analysis of mega-telomeres signals was standardized using techniques described previously [24,28]. Forty to 50 cells were examined for each FISH experiment for a total of more than 4, 400 cells analyzed.

## Results

### Characterization of MDV Integration Sites

**Figure 1:**
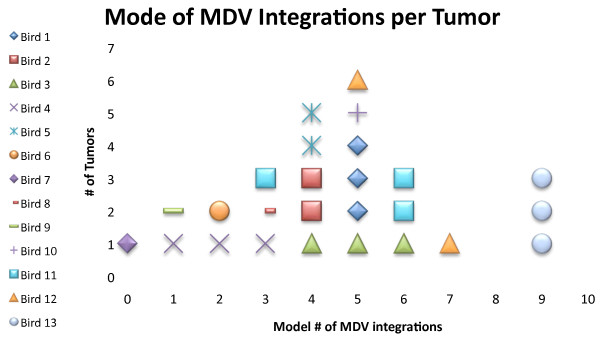
**Distribution of the modal number of MDV integrations per lymphoma**. MDV integration sites were analyzed in 26 tumors from 13 birds and the mode was calculated for each tumor. The overall mode was five integrations, with a range of zero (mean in this tumor was less than one integration, see Table 1) to nine. Each symbol in the diagram represents one tumor, and tumors within a bird are the same color and symbol or shape.

The status of MDV integrations was studied by profiling 26 tumors from 13 birds; integrations were observed in every lymphoma examined (Table 2). The overall mode of integration sites was 5 (Figure 1) and the overall mean for number of viral integration sites was 4.17 (range 0.2 to 8.0). Several control experiments were conducted to aid in MDV signal interpretation including hybridization of the MDV BAC probe to samples from non-infected birds (adults and embryos) and hybridization of two MDV cosmid clones to tumor samples. In the first case, no virus signal was detected in non-infected individuals (Additional Figure 1: Hybridization of the MDV BAC probe to uninfected embryonic chicken cells). In the second case, overlapping MDV cosmid clones, P89 and SN16, used because they lacked telomeric repeats [21,29], were hybridized against a previously profiled tumor sample (Additional Figure 2: FISH comparison between two probes: the MDV-BAC and MDV cosmid clones). The mode of MDV integrations was found to be the same as when hybridized with the full-genome MDV BAC. Thus, these controls demonstrated that the MDV BAC probe was specific for hybridization to only the MDV DNA versus any elements of the chicken genome.

**Table 2:** A cytogenomic profile of lymphomas from MDV-infected birds: virus integrations, ploidy, and telomeres.

Sample Information			MDV Integration Sites*			**Ploidy Levels (metaphase cells)**^†^		**Mega-Telomere Signals**^‡^	
Bird #	Sex	Tumor # (Tissue Source)	Mode	Mean	SD	% Tetraploid	% Aneuploid	Mode	W
1	M	1-T1 (heart)	5	4.4	0.9	2	0	7	-
		1-T2 (left gonad)	5	5.4	1.3	0	0	11	-
		1-T3 (lung)	5	4.9	0.7	0	0	9	-
2	M	2-T1 (left gonad)	4	3.8	0.7	4	0	7	-
		2-T2 (right gonad)	4	4.1	1.1	4.5	0	7	-
3	M	3-T1 (lung)	4	4.7	1.6	4	0	6	-
		3-T2 (bursa)	5	4.3	1.3	16	0	7	-
		3-T3 (gonad)	6	5.9	0.8	5	0	6	-
4	F	4-T1 (spleen 1)	3	3.2	1.2	0	0	9	Yes
		4-T2 (spleen 2)	2	1.9	1.2	0	0	7	Yes
		4-T3 (heart)	1	1.4	1.3	0	0	7	Yes
5	M	5-T1 (left gonad)	4	3.8	0.8	0	0	8	-
		5-T2 (right gonad)	4	3.6	0.8	6	0	8	-
6	M	6-T1 (heart)	2	1.6	1.2	0	0	7	-
7	F	7-T1 (spleen)	0	0.2	0.6	0	0	8	Yes
8	F	8-T1 (gonad)	3	2.4	1.4	0	0	10	Yes
9	F	9-T1 (liver)	1	0.5	0.5	0	0	8	Yes
10^§^	F	10-T1 (gonad)	5	5.0	1.0	25	12.5	9	Yes
11^§^	M	11-T1 (kidney)	3	3.5	2.2	21	23	5	-
		11-T2 (liver)	6	4.5	2.2	17.5	15	7	-
		11-T3 (thymus)	6	5.4	1.3	20	2.5	8	-
12^§^	F	12-T1 (gonad)	5	5.6	2.0	27	7	6	Yes
		12-T2 (kidney)	7	5.5	2.2	0	0	6	-^¶^
13^§^	F	13-T1 (spleen)	9	7.2	3.0	2.5	0	7	Yes
		13-T2 (liver)	9	8.0	1.8	0	10	7	Yes
		13-T3 (kidney)	9	7.7	2.1	10	7	7	Yes

*Mode (most frequent value), mean, and standard deviation (SD) of diploid metaphase cells.

^†^Tetraploid cells were identified as those containing four copies of all macrochromosomes and sex chromosomes, and an estimated number of microchromosomes present in twice the normal amount. Aneuploid cells were identified as those containing an abnormal copy number (less than or greater than two) of one or more macrochromosomes.

^‡^Mega-telomere signals were counted in tumor metaphase cells using the telomere-PNA probe, and the mode was calculated for each tumor.

^§^Birds from infection strategy 2 (see Methods section for details).

^¶^Tumor 12-T2 lacked a W chromosome (as verified by the W BAC probe, see Table 1).

**Figure 2:**
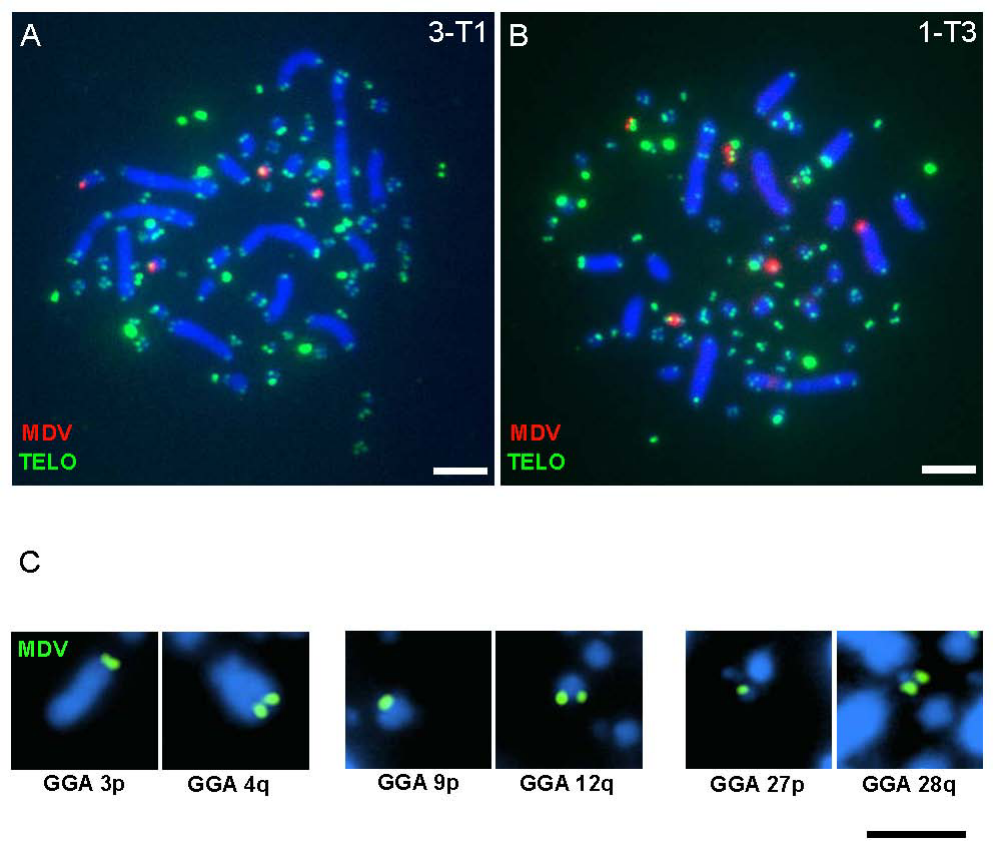
**Localization of MDV integrations at the telomeres of chicken chromosomes**. Panels **A **and **B **show representative results of the MDV integrations (*red*) observed at the telomeres (*green) *of the chicken genome of MDV-induced lymphomas. Panel **C **shows representative chromosomes with MDV integrations (*green*) to illustrate that integrations were observed in all size-categories of chromosomes (both macro-and microchromosomes) and there was no preference for p or q arm telomere. Panels **A **and **B **also illustrate the telomeric array profile observed in the tumors from 15I_5 _X 7_1_, the heterozygous cross used in this study. For each image, the telomeric signals were adjusted consistently by adjusting GGA 1 interstitial signals so that they were still visible, while not over-saturating the mega-telomere arrays. Chromosomes are blue (DAPI), the telomeres are green (FITC) and the MDV DNA is red (rhodamine, panels **A **and **B**) or green (FITC, panel **C**), see Methods. *Scale bar*, 5 μm

In all tumors examined, the MDV integrations were localized at the telomeres (Figure 2) and interstitial chromosome integration was not observed on the macrochromosomes or the microchromosomes, to the extent allowed by cytogenetic resolution. When sister chromatids were visibly separated [22], two MDV signals were observed, one on each sister chromatid. Only one integration site per chromosome was observed, i.e., MDV was only found at either the p or the q arm of the involved chromosome, but not at both arms. Chromosomal p versus q arm telomere integration was easily determined in the macrochromosomes (GGA 1-5) based on cytogenetic observations (e.g., GGA 3p and 4q, Figure 2C). The p versus q arm telomere integrations into intermediate (GGA 6-10) and microchromosomes (GGA 11-28) were established using cytogenetic observations and the sequenced genome assembly location for the chromosome-specific BAC probes used in the mapping experiments. For example, see GGA 9p, 12q, 27p and 28q in Figure 2C. For a few of the smallest microchromosomes, the p versus q arm position of MDV integration could not be established because BAC assembly information and sequence coordinates were not available.

### Identification of Involved Chromosomes by FISH Mapping

**Figure 3:**
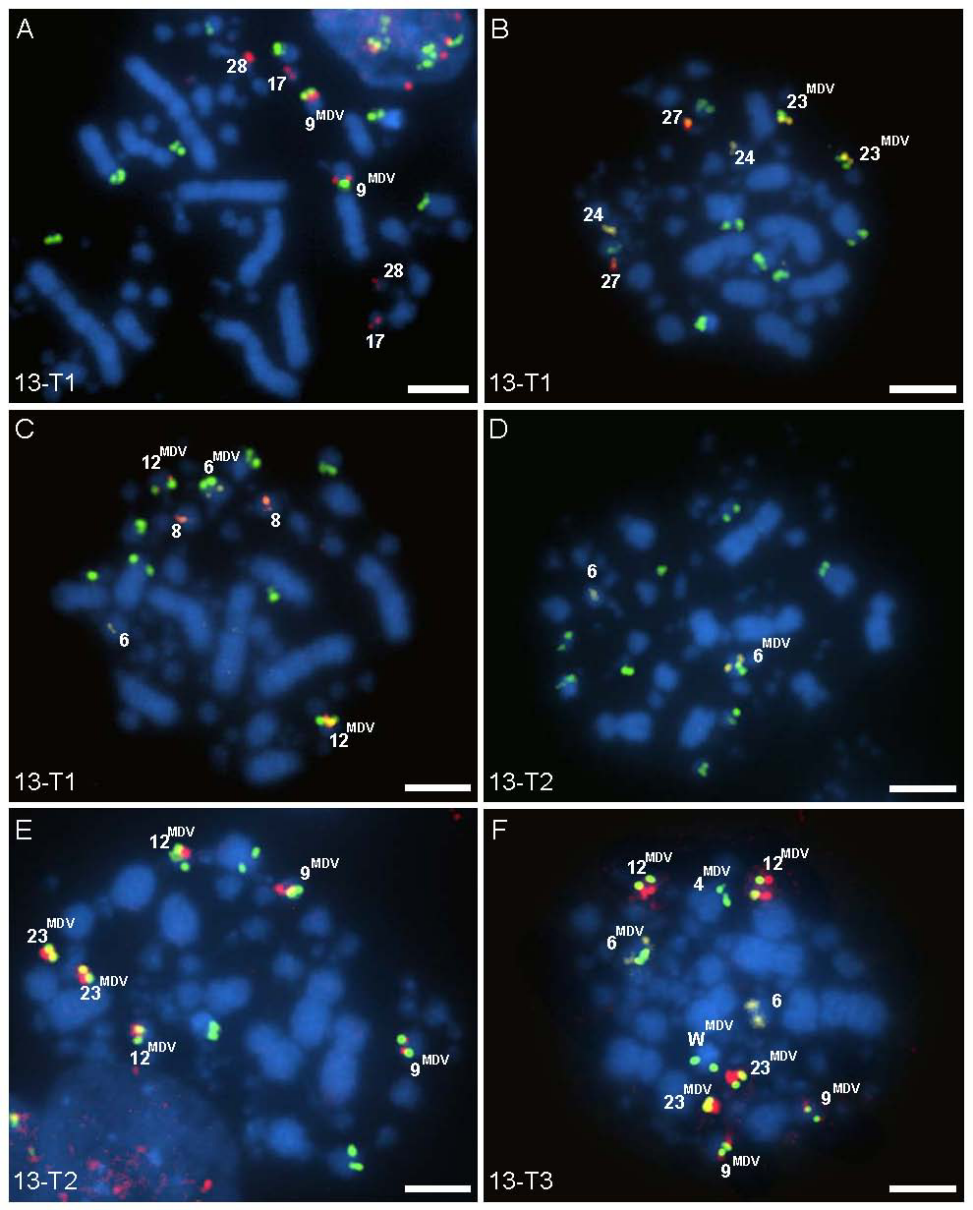
**Mapping of MDV integrations in chicken chromosomes**. An iterative process of mapping by multicolor FISH was used to identify the involved chromosomes. Images shown are from Bird 13, which had nine integration sites (4p, 6p, 9q, 9q, 12, 12, 23, 23, and Wp) for all three tumors. Cells shown in **A, B**, and **C **are from tumor 13-T1, which was used to begin mapping. Once the positive sites were identified for 13-T1, they were subsequently tested on 13-T2 **(D **and **E) **and 13-T3 **(F)**. Multiple chromosomal BAC probes were tested for possible integration in panels **A**-**C**. The MDV positive chromosomes are indicated by superscript. Chromosomal BAC probes are labeled either red (Spectrum Red) or yellow (Spectrum Orange) and in all panels MDV DNA is labeled green (FITC), see Methods. *Scale bar*, 5 μm

Chromosome-specific BACs were utilized (Table 1) to identify the specific chicken chromosomes harboring MDV integrations. Our approach was to hybridize more than one chromosomal BAC (~ three at a time) to one of the tumor samples for a bird (e.g., 13-T1, Figure 3A, B, and 3C) and any dual-signal positive chromosomes were recorded (e.g., GGA 9, Figure 3A). The entire suite of chromosomal BACs was tested on the same tumor until all of the MDV integrations had been identified or until all available BACs were tested. Next, the positive chromosomal BACs were hybridized to the other tumor samples from that bird (e.g., 13-T2 and 13-T3, Figure 3D,E,a). If the tumors showed different integration profiles then each tumor was tested separately with all available BACs. Integrations in fifteen tumors from six birds were mapped using this iterative approach and the results are found in Table 3. Some viral integration positions remain "unknown" due to the lack of sequence assembly information and BAC probes for microchromosomes (GGA 29-38).

**Table 3:** Chromosome locations of MDV integrations in lymphomas: Telomeres of autosomes and sex chromosomes.

Tumor Identity*	% of cells	Chromosomes with Integrations	# of Unmapped Integration Sites^†^
1-T1	100	3p, 23, 24p	2
1-T2	68	3p, 23, 24p	2
"	32	1q, 4q, 8p, 9p, 9p, 20, 28q, Z	0
1-T3	87	3p, 23, 24p	2
"	13	1q, 4q, 8p, 9p, 9p, 20, 28q, Z	0
			
2-T1	100	15q, 17q, 20q, 20q	0
2-T2	100	15q, 17q, 20q, 20q	0
			
3-T1	100	10, 11, 12q, 18	0
3-T2	100	10, 11, 12q, 13, 18	0
3-T3	100	4q, 6p, 12q, 18, 19p	1
			
5-T1	100	7q, 12q, 20q, 27p	0
5-T2	100	7q, 12q, 20q, 27p	0
			
12-T1	100	12, 26	3
12-T2	74	3p, 6p, 24, 25	3
"	26	none	2
			
13-T1	100	4p, 6p, 9q, 9q, 12, 12, 23, 23, Wp	0
13-T2	100	4p, 6p, 9q, 9q, 12, 12, 23, 23, Wp	0
13-T3	100	4p, 6p, 9q, 9q, 12, 12, 23, 23, Wp	0

*See Table 2 for additional tumor sample information (Bird #, sex, and tissue source).

**^†^**Unidentified chromosomes with MDV integration are microchromosomes (GGA 29-38) that lacked assembled sequence information or BAC probes at the time of this study.

In terms of the uniformity of integration positions among tumors from a single individual, four different categories of results were observed. First, three individuals (Birds 2, 5, and 13) each had multiple tumors that showed complete uniformity among tumors. Figure 3 is a composite of FISH images from three tumors from one individual (Bird 13) each showing nine identical integration sites and provides a representative of the iterative process of mapping the involved chromosomes. The second category of results observed was heterogeneity within a tumor, e.g., the Bird 1 profile. Two of three tumors from Bird 1 (1-T2 and 1-T3) included sub-populations of cells with integration sites differing from the main population of cells (which were shared). The sub-populations of each tumor shared the same profile with eight total integration sites, different from the main profile, which had five integration sites (Table 3). The third results category was observed in Bird 3, having in-common sites in each of its three tumors (3-T1, 3-T2, and 3-T3) but also unique integration sites in each tumor. Tumors 3-T1 and 3-T2 had four in-common locations at GGA 10, 11, 12, and 18 and tumor 3-T2 contained an additional integration site at GGA 13. Tumor 3-T3 had two in-common sites with both 3-T1 and 3-T2 (GGA 12 and 18); however, it also contained four unique sites. And finally, the fourth results category was found in Bird 12, which was the only individual with two tumors (12-T1 and 12-T2) that had no in-common chromosomal integration sites. Tumor 12-T1 displayed a uniform integration profile, which differed from the main profile of 12-T2. In addition, 12-T2 had a subpopulation of cells which had two integration sites which could not be mapped (lack of probes and assembly information) (Table 3).

### Karyotype Abnormalities and Mega-telomere Loci

**Figure 4:**
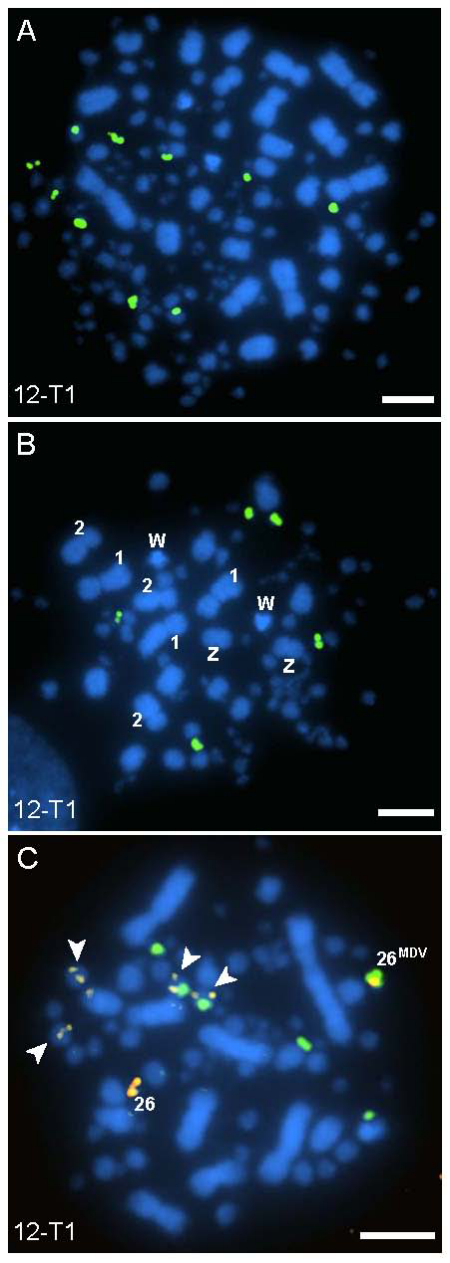
**MDV-induced lymphomas exhibit a variety of ploidy-level abnormalities**. Karyotype abnormalities were common in the tumors and the tetraploid cells typically had double the number of MDV integration sites (*green*) as compared to diploid cells. As shown here, a tetraploid cell **(A) **from tumor 12-T1 of Bird 12 had ten MDV integration sites (the majority of diploid cells for 12-T1 had five integration sites). Aneuploid cells **(B **and **C) **were also observed in the tumors, in the cell shown in panel **B **there are three copies of GGA 1 and 2, and two copies of both GGA W and Z. The cell shown in panel **C **from 12-T1 has several karyotype abnormalities, including four copies of GGA 12 (arrowheads, *yellow *signals), two of which have MDV on them. The mode of integrations for diploid cells in 12-T1 was five, where as the cell in panel **C **displays six MDV integration sites with the addition of the tetrasomic GGA 12, indicating non-disjunction occurred after viral integration. GGA 26 (*red) *is also shown here as having one homolog positive for MDV integration (indicated by superscript text). *Scale bar*, 5 μm

Several karyotype anomalies were found in the tumors, a phenomenon well established in human cancers. Variations included ploidy changes, commonly tetraploid and aneuploid (Figure 4). The tetraploid cells typically contained double the number of MDV integrations of the diploid cells suggesting the ploidy change occurred after integration.

We investigated the telomere profile of tumors cells to consider whether there was an observable preference for integration into the extremely large (aka mega-) telomeres of the chicken genome, see Figure 2A,B and Table 2. The number of mega-telomeres ranged from 5 to 11 among individuals and intra-individual diversity was observed. For example, Bird 1 indicated different mega-telomere profiles in each of its tumors with modes of 7 (1-T1), 9 (1-T3) and 11 (1-T2). As reported in prior work (36), the females exhibited an extremely large (mega-) telomere on the W sex chromosome. However, there was no observable preference for MDV integration into the mega-telomere loci.

## Discussion

Delecluse and colleagues provided initial evidence for viral integration of MDV into chicken chromosomes [30,31]. At the time, this work was necessarily limited in scope and resolution by the lack of sequence information and probes for both the MDV and the chicken chromosomes. Having the advantage of genomics tools and high resolution cytogenetics, we are able to now show that the MDV genome is present in an integrated state at the telomeres of specific chicken chromosomes of tumors resulting from MDV-infection. MDV was observed on both sister chromatids indicating the viral DNA replicates in a semi-conservative fashion along with the host genomic DNA. We also noted that signal intensity varied among integration locations, suggestive of different numbers of integrated copies. The sequencing of the chicken genome coupled with the availability of chromosome specific BACs to identify chicken microchromosomes [24,25,32] created the opportunity for mapping of integrations and allowed us to address several crucial questions related to integration site preference and lineages of transformation events.

### Preferences of Viral Integration: Subchromosomal and Chromosomal

#### MDV integrates at the chicken telomeres

Our initial working hypothesis was that MDV preferentially integrates into the chicken mega-telomeres, which are ultra-long telomeric arrays ranging in size from 100 s of Kb to several Mb [24,28,33-35]. Although our data did not support this hypothesis, the MDV genome was found exclusively at the telomeric regions of the host chromosomes. Viral integration into the interstitial telomeric sequences was not observed. We hypothesize that the terminal ends of chromosomes may provide an environment more conducive to integration, perhaps through a specialized chromatin confirmation (e.g., the t-loop of the telomere as it is replicated, and/or the actual telomerase extension apparatus). Interestingly, HHV-6 was found integrated into the subtelomeric region of human chromosomes and, similar to MDV, possesses telomere repeat arrays within its genome [9,36]. The telomeric array repeats were hypothesized to assist the HHV-6 integration mechanism through homologous recombination [9].

In exploring our initial hypothesis regarding MDV preference for integration into mega-telomeres we found additional evidence for extensive genotypic variation in telomere array sizes within and among chicken genetic lines and cell lines [28,35]. Although variation for the number of mega-telomere loci observed among the birds in this study was to be expected from an F1 generation (see methods), the variation among tumors within birds was not expected. Inter-tumor variation (within an individual) could be a manifestation of gain or loss of telomeric repeat sequence due to genomic instability. Another consideration is the unknown effect that integration of MDV DNA may have upon the telomere sequence itself.

#### MDV integration: random versus targeted?

**Figure 5:**
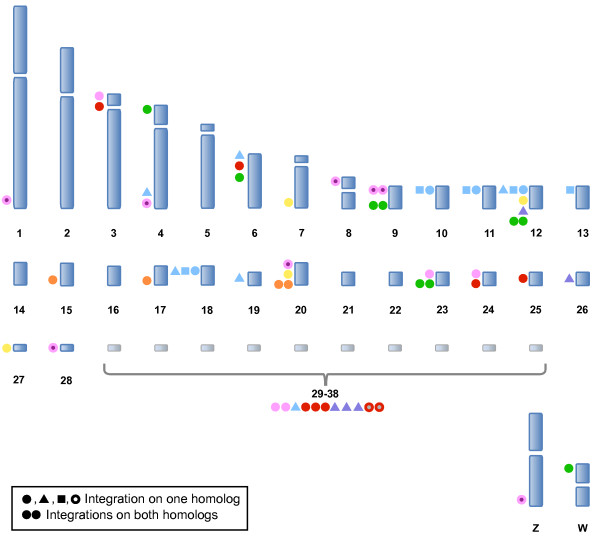
**MDV integration site preference: Distribution across chicken chromosomes**. To determine if MDV preferentially integrated into a specific set of host chromosomes, integration sites for 15 tumors were mapped and analyzed. Chromosomes 4, 6, 12, and 20 are noteworthy as they are shared integration sites among three or more birds. Each tumor is represented by a different color and symbol, which correspond to the tumors depicted in Figure 6 for each bird mapped. Symbols are located to the left of the chromosome for which integration was mapped. If multiple tumors from one bird all share identical integration sites (e.g. Bird 13) then only one set of symbols (e.g. green circles) represents the single transformation event that occurred. If multiple tumors from one bird each have different integration profiles (e.g. Bird 3) then multiple shapes and/or colors (e.g. light blue circles, squares, and triangles) were used. Finally, if a derived cell population was present within a tumor (e.g. Bird 1, T2 and T3) then the sites representing the derived population are depicted as circles outlined in the main tumor color and filled in with a different color (e.g. on GGA 9, 1-T2 and 1-T3).

Unlike HHV-6, which typically integrates into one or two chromosomes [10,14,37] MDV integrates into more chromosomes, typically five (and as many as nine). We found integrations across all size categories of chromosomes, i.e., both macro-and microchromosomes (Figure 2C). A few chromosomes stand out as interesting candidates for future studies, as highlighted by Figure 5. In the two instances that MDV was found integrated on GGA 9, both homologs were involved. GGA 9 encodes the single copy chicken telomerase RNA (cTR) component of the telomerase enzyme [36]. Sequencing of the MDV genome showed that it also encodes two copies of TR (viral (v)TR) with high sequence identity (85-88%) to cTR [16,17,38,39]. In comparison to cTR, vTR was more efficient at restoring telomerase activity in telomerase negative cell systems [39]. Furthermore, when vTR was deleted from the MDV genome, the vTR-deleted virus showed reduced capabilities in causing lymphomas, suggesting that vTR is a key element in promoting T-cell lymphomagenesis [40]. Whether MDV is specifically utilizing vTR to aid in integration by taking advantage of telomerase activity at the telomeres requires further study, and would be a novel role for TR (chicken or viral).

Several other chromosomes frequently exhibited MDV integrations, for example GGA 4, 6, 12, and 20, were found in common among tumors between three or more birds (Figure 5). Interestingly, GGA 4 encodes the chemokine interleukin-8 (IL-8) gene sequence [41] and MDV sequencing showed the virus also encodes an IL-8 gene [16,42]. The presence of host sequences in the virus genome is noteworthy because a plausible mechanism for MDV to obtain such chicken-derived genes (vTR and vIL-8) and sequences (e.g., TTAGGG repeats) is by integration into the chicken chromosomes, and then subsequent emergence with exogenous host sequence. Since telomeric integration appears to be the preferred location, we propose that viral integration near host genes is a rare event. However, if a "captured" host gene sequence provides a selective advantage to the virus, e.g., vTR's ability to promote oncogenesis or perhaps facilitate integration, it would persist in future generations of virus.

### Clonality of Late-Stage Lymphomas: Lineage relationships among tumors within birds

Another objective of this study was to improve our understanding regarding clonality of MDV-induced lymphomas. That is, are the multiple tumors of a bird derived from a single transformation event (monoclonal origin) or do the tumors arise from independent events (polyclonal origin)? Clonality of tumors has been addressed by immunoglobulin analysis, T-cell receptor gene rearrangements, X-chromosome methylation patterns, various molecular markers for somatic mutations, and analysis of viral integration [reviewed in [43-48]]. Viral integration sites and viral sequencing have proved to be useful markers when assessing clonality of tumors [49]. Here we utilized the mapped MDV integrations to gain insight into tumor lineage(s).

**Figure 6:**
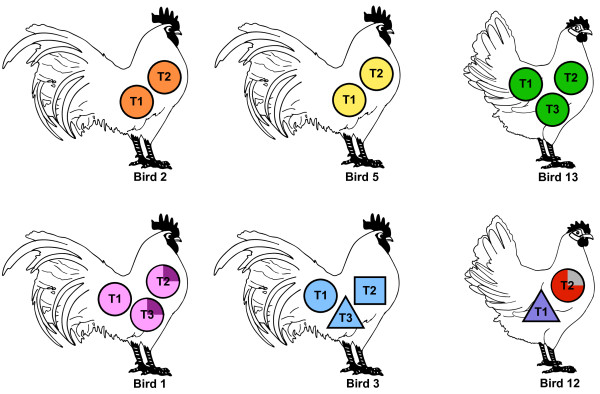
**Lineage analysis by mapping of MDV-induced T-cell lymphomas indicates a predominately, but not exclusively, monoclonal origin**. The mapped MDV integration sites of 15 tumors from 6 birds (4 males and 2 females) provide evidence for several different modes of clonality, predominant being monoclonal. Four of the birds (Bird 1, 2, 5, and 13) displayed tumors indicating a monoclonal origin, and one of the four (Bird 1) exhibited a derived cell population. Tumors from Bird 3 possessed both in-common integration sites and unique sites for each tumor, potentially indicating a polyclonal origin. Bird 12 also displayed two tumors with a polyclonal origin, and a derived cell population in 12-T2. Both tumors from Bird 12 also have three unidentified integration sites and they could possibly be in-common sites. Each tumor is represented by a colored shape, with the tumor number indicated (see Table 3), which corresponds to the symbols found in Figure 5. The same shape and color tumor indicates identical integration sites between those tumors. Tumors that contain both in-common and unique sites (Bird 3) are represented with different shapes and the same color. If a tumor exhibited a derived cell population (1-T2, 1-T3, and 12-T2) it is depicted as a quarter section of the circle filled with a different color. Those tumors with no in-common sites (Bird 12) are represented by a different color and shape.

Mapping lineage analysis indicates that four (of six) birds had multiple tumors of monoclonal origin, inclusive of one individual (Bird 1) showing a derived cell population (Figure 6), which is not unexpected given lack of genome stability in cancers and/or the possibility that the virus could be mobile. The remaining two birds (Bird 3 and 12) displayed tumors consist with a mixed or polyclonal origin (Figure 6). The most conservative analysis of the three tumors from Bird 3 places them in a polyclonal category, that is, three separate transformation events occurred to form each tumor. Alternatively, the tumors from Bird 3 could be related, which raises the possibility that the virus is capable of remerging from its integrated state and moving to new integrated locations. Alternatively, tumors 3-T1 and 3T-2 could be of monoclonal origin given the loss of one of the integration sites (GGA 13). However, the third tumor (3-T3) showed only two MDV integration sites (GGA 12 and 18) in common with the other two tumors, suggestive of a separate transformation event. The tumors from Bird 12 may also represent a polyclonal set of tumors, indicating tumors 12-T1 and 12-T2 have different founder cells altogether (Figure 6). However, it must be noted that 12-T1 and 12-T2 both have three unidentified MDV integration sites and the possibility remains that those three sites could be in common.

## Conclusions

The indications for predominately, but not exclusively, monoclonal tumor origins, as well as derived cell populations within the MDV lymphomas, illustrates the complexity of tumor biology and the potentially diverse strategies employed by the herpesvirus. Understanding the timeframe during pathogenesis when MDV integrates into the chicken genome will be essential to assessing both the utility of integration profiles as a marker for clonality and the significance of integration on the latency cycle. In addition, further investigation of the effect of viral integration on cellular transformation is necessary. Integration may have a profound effect on transformation, or it may simply serve as a mechanism of maintaining the latent genome. We are currently investigating the stages at which MDV integrates, from early infection to late oncogenesis, and have preliminary indications that MDV integrates prior to transformation and tumorigenesis. Further research on the molecular mechanism for telomere-targeting of MDV integration should provide new insight regarding the role of telomeres in aspects of host genome stability and also viral evolution.

## Competing interests

The authors declare that they have no competing interests.

## Authors' contributions

CMR, HDH, HHC, and MED conceived of and designed the research project. CMR conducted the experiments. CMR and MED wrote the manuscript. All authors have read and approved the final manuscript.

## Supplementary Material

Additional file 1 **Hybridization of the MDV BAC probe to uninfected embryonic chicken cells**. The MDV BAC was hybridized to chromosomes from uninfected chicken embryo fibroblasts. As shown, the MDV BAC probe did not hybridize to uninfected chicken cells, i.e., there are no FISH signals observed. *Scale bar*, 5 μm Click here for file

Additional file 2 **FISH comparison between two probes: the MDV-BAC and MDV cosmid clones**. Panel **A **displays tumor 10-T1 labeled with the MDV cosmid clones (*green) *with a total of five integration sites indicated by arrowheads. Panel **B **displays tumor 10-T1 labeled with the full-genome MDV BAC probe (*red) *also with a total of five integration sites. *Scale bar*, 5 μm Click here for file

## References

[B1] Mettenleiter TC Keil GM Fuchs W Mettenleiter TC, Sobrino F Molecular biology of animal herpesviruses Animal Viruses: Molecular Biology 2008 Norfolk UK: Caister Academic Press 375 455

[B2] Batista FM Arzul I Pepin JF Ruano F Friedman CS Boudry P Renault T Detection of ostreid herpesvirus 1 DNA by PCR in bivalve mollusks: a critical review J Virol Methods 2007 139 1 11 10.1016/j.jviromet.2006.09.005 17079025

[B3] Marek J Multiple Nervenentzuendung (Polyneuritis) bei Huehnern Deutsche Tierärztliche Wochenschrift 1907 15 417 421

[B4] Schat KA Nair V Saif YM, Fadly AM, Glisson JR, McDougald LR, Nolan LK, Swayne DE Marek's Disease Diseases of Poultry 2008 12 Ames IA: Iowa State Univ. Press 452 514

[B5] Osterrieder N Kamil JP Schumacher D Tischer BK Trapp S Marek's disease virus: from miasma to model Nat Rev Micro 2006 4 283 294 10.1038/nrmicro1382 16541136

[B6] Witter RL Protective efficacy of Marek's disease vaccines Curr Topics Microbiol Immunol 2001 255 57 90 10.1007/978-3-642-56863-3_3 11217428

[B7] Witter RL Increased virulence of Marek's disease virus field isolates Avian Dis 1997 41 149 163 10.2307/1592455 9087332

[B8] Morissette G Flamand L Herpesviruses and chromosomal integration Journal of Virology 2010 84 12100 12109 10.1128/JVI.01169-10 20844040 PMC2976420

[B9] Arbuckle JH Medveczky MM Luka J Hadley SH Luegmayr A Ablashi D Lund TC Tolar J De Meirleir K Montoya JG Komaroff AL Ambros PF Medveczky PG The latent human herpesvirus-6A genome specifically integrates in telomeres of human chromosomes in vivo and in vitro Proc Natl Acad Sci USA 2010 107 5563 5568 10.1073/pnas.0913586107 20212114 PMC2851814

[B10] Hall CB Caserta MT Schnabel K Shelley LM Marino AS Carnahan JA Yoo C Lofthus GK McDermott MP Chromosomal integration of human herpesvirus 6 is the major mode of congenital human herpesvirus 6 infection Pediatrics 2008 122 513 520 10.1542/peds.2007-2838 18762520

[B11] Gao J Luo X Tang K Li X Guiyuan L Epstein-Barr virus integrates frequently into chromosome 4q, 2q, 1q, and 7q of Burkitt's lymphoma cell line (Raji) Journal of Virological Methods 2006 136 193 199 10.1016/j.jviromet.2006.05.013 16806502

[B12] Reisinger J Rumpler S Lion T Ambros PF Visualization of episomal and integrated Epstein-Barr virus DNA by fiber fluorescence *in situ *hybridization Int J Cancer 2006 118 1603 1608 10.1002/ijc.21498 16217752

[B13] Morris C Luppi M McDonald M Barozzi P Torelli G Fine mapping of an apparently targeted latent human herpesvirus type 6 integration site in chromosome band 17p13.3 J Med Virol 1999 58 69 75 10.1002/(SICI)1096-9071(199905)58:1<69::AID-JMV11>3.0.CO;2-3 10223549

[B14] Daibata M Taguchi T Taguchi H Miyoshi I Integration of human herpesvirus 6 in Burkitt's lymphoma cell line J Haematol 1998 102 1307 1313 10.1046/j.1365-2141.1998.00903.x 9753061

[B15] Izumiya Y Janng H-K Ono M Mikami T Hirai K A complete genome sequence of Marek's disease virus type 2, strain HPRS24 Marek's disease virus. Curr Topics Microbiol Immunol 2001 255 New York: Springer 25 55 10.1007/978-3-642-56863-3_8 11217423

[B16] Lee LF Wu P Sui D Ren D Kamil J Kung HJ Witter RL The complete unique long sequence and the overall genomic organization of the GA strain of Marek's disease virus Proc Natl Acad Sci USA 2000 97 6091 6096 10.1073/pnas.97.11.6091 10823954 PMC18563

[B17] Tulman ER Afonso CL Lu Z Zsak L Rock DL Kutish GF The genome of a very virulent Marek's disease virus Journal of Virology 2000 74 7980 7988 10.1128/JVI.74.17.7980-7988.2000 10933706 PMC112329

[B18] Niikura M Dodgson J Cheng H Direct evidence of host genome acquisition by the alphaherpesvirus Marek's disease virus Arch Virol 2006 151 537 549 10.1007/s00705-005-0633-7 16155725

[B19] Petherbridge L Brown AC Baignet SJ Howes K Sacco MA Osterrieder N Nair VK Oncogenicity of virulent Marek's disease virus cloned as bacterial artificial chromosomes Journal of Virology 2004 78 13376 13380 10.1128/JVI.78.23.13376-13380.2004 15542691 PMC525015

[B20] Petherbridge L Howes K Baigent SJ Sacco MA Evans S Osterrieder N Nair V Replication-competent bacterial artificial chromosomes of Marek's disease virus: novel tools for generation of molecularly defined herpesvirus vaccines Journal of Virology 2003 77 8712 8718 10.1128/JVI.77.16.8712-8718.2003 12885890 PMC167215

[B21] Kishi M Bradley G Jessip J Tanaka A Nonoyama M Inverted repeat regions of Marek's disease virus DNA possess a structure similar to that of the a sequence of herpes simplex virus DNA and contain host cell telomere sequences Journal of Virology 1991 65 2791 2797 1851854 10.1128/jvi.65.6.2791-2797.1991 PMC240894

[B22] Bacon LD Hunt HD Cheng HH A review of the development of chicken lines to resolve genes determining resistance to diseases Poultry Sci 2000 79 1082 1093 10.1093/ps/79.8.1082 10947175

[B23] Romanov MN Daniels LM Dodgson JB Delany ME Integration of the cytogenetic and physical maps of chicken chromosome 17 Chromosome Res 2005 13 215 222 10.1007/s10577-005-1506-3 15861310

[B24] Delany ME Gessaro TM Rodrigue KL Daniels LM Chromosomal mapping of chicken mega-telomere arrays to GGA 9, 16, 28 and W using a cytogenetic approach Cytogenet Genome Res 2007 117 54 63 10.1159/000103165 17675845

[B25] McMillan D Miethke P Alsop AE Rens W O'Brien P Trifonov V Veyrunes F Schatzkamer K Kremitzki CL Graves T Warren W Grützner F Ferguson-Smith MA Marshall Graves JA Characterizing the chromosomes of the platypus (Ornithorhynchus anatinus) Chromosome Res 2007 15 961 974 10.1007/s10577-007-1186-2 18185982

[B26] Lee MK Ren CW Yan B Cox B Zhang HB Romanov MN Sizemore FG Suchyta SP Peters E Dodgson JB Construction and characterization of three BAC libraries for analysis of the chicken genome Anim Genet 2003 34 151 152 10.1046/j.1365-2052.2003.00965_5.x 12648103

[B27] Delany ME Krupkin AB Molecular characterization of ribosomal gene variation within and among NORs segregating in specialized populations of chicken Genome 1999 42 60 71 10.1139/gen-42-1-60 10208002

[B28] O'Hare TH Delany ME Genetic variation exists for telomeric array organization within and among the genomes of normal, immortalized, and transformed chicken systems Chromosome Res 2009 17 947 964 19890728 10.1007/s10577-009-9082-6 PMC2793383

[B29] Reddy SM Lupiani B Gimeno IM Silva RF Lee LF Witter RL Rescue of a pathogenic Marek's disease virus with overlapping cosmid DNAs: use of a pp38 mutant to validate the technology for the study of gene function Proc Natl Acad Sci USA 2002 99 7054 7059 10.1073/pnas.092152699 11997455 PMC124527

[B30] Delecluse H Hammerschmidt W Status of Marek's disease virus in established lymphoma cell ilnes: herpesvirus integration is common Virology 1993 67 82 92 10.1128/jvi.67.1.82-92.1993 PMC237340 8380099

[B31] Delecluse HJ Hammerschmidt W Latent Marek's disease virus can be activated from its chromosomally integrated state in herpesvirus-transformed lymphoma cells EMBO 12 3277 3286 10.1002/j.1460-2075.1993.tb05997.x PMC413595 8393785

[B32] Ren C Lee MK Yan B Ding K Cox B Romanov MN Price JA Dodgson JB Zhang HB A BAC-based physical map of the chicken genome Genome Res 2003 13 2754 2758 10.1101/gr.1499303 14656976 PMC403818

[B33] Delany ME Krupkin AB Miller MM Organization of telomere sequences in birds: evidence for arrays of extreme length and for in vivo shortening Cytogenet Cell Genet 2000 90 139 145 10.1159/000015649 11060464

[B34] Nanda I Schmid M Localization of the telomeric (TTAGGG)_n _sequence in chicken (*Gallus domesticus) *chromosomes Cytogenet Cell Genet 1994 65 190 193 10.1159/000133630 8222759

[B35] Rodrigue KL May BP Famula TR Delany ME Meiotic instability of chicken ultra-long telomeres and mapping of a 2.8 megabase array to the W-sex chromosome Chromosome Res 2005 13 581 591 10.1007/s10577-005-0984-7 16170623

[B36] Gompels UA Macaulay HA Characterization of human telomeric repeat sequences from human herpesvirus 6 and relationship to replication J Gen Virol 1995 76 451 458 10.1099/0022-1317-76-2-451 7844567

[B37] Nacheva EP Ward KN Brazma D Virgili A Howard J Leong HN Clark DA Human herpesvirus 6 integrates within telomeric regions as evidenced by five different chromosomal sites J Med Virol 2008 80 1952 1958 10.1002/jmv.21299 18814270

[B38] Delany ME Daniels LM The chicken telomerase RNA gene: conservation of sequence, regulatory elements and synteny among viral, avian, and mammalian genomes Cytogenet Genome Res 2003 102 309 317 10.1159/000075768 14970722

[B39] Fragnet L Blasco MA Klapper W Rasschaert D The RNA subunit of telomerase is encoded by Marek's disease virus Journal of Virology 2003 77 5985 5996 10.1128/JVI.77.10.5985-5996.2003 12719590 PMC154048

[B40] Trapp S Parcells MS Kamil JP Schumacher D Tischer BK Kumar PM Nair VK Osterrieder N A virus-encoded telomerase RNA promotes malignant T cell lymphomagenesis J Exp Med 2006 203 1307 1317 10.1084/jem.20052240 16651385 PMC2121211

[B41] Kaiser P Hughes S Bumstead N The chicken 9E3/CEF4 CXC chemokine is the avian orthologue of IL8 and maps to chicken chromosome 4 syntenic with genes flanking the mammalian chemokine cluster Immunogenetics 1999 49 673 684 10.1007/s002510050664 10369926

[B42] Parcells MS Marek's disease virus (MDV) encodes an Interleukin-8 homolog (vIL-8): characterization of the vIL-8 protein and a vIL-8 deletion mutant MDV Journal of Virology 2001 75 5159 5173 10.1128/JVI.75.11.5159-5173.2001 11333897 PMC114921

[B43] Leedham SJ Wright NA Human tumour clonality assessment-flawed but necessary J Pathol 2008 215 351 354 10.1002/path.2379 18566960

[B44] Wainscoat JS Fey MF Assessment of clonality in human tumors: a review Cancer Research 1990 50 1355 1360 1967978

[B45] Fey MF Wainscoat JS Molecular diagnosis of haematological neoplasms Blood Reviews 1988 2 78 87 10.1016/0268-960X(88)90028-8 3042060

[B46] Arnold A Cossman J Bakhshi A Jaffe ES Waldmann TA Korsmeyer SJ Immunoglobulin-gene rearrangements as unique clonal markers in human lymphoid neoplasms New England Journal of Medicine 1983 309 1593 1599 10.1056/NEJM198312293092601 6417538

[B47] Baikie AG Court-Brown WM Buckton KE Harnden DG Jacobs PA Tough IM A possible specific chromosome abnormality in human chronic myeloid leukaemia Nature 1960 188 1165 1166 10.1038/1881165a0 13685929

[B48] Nowell PC Hungerford DA Chromosome studies on normal and leukemic human leukocytes J Natl Cancer Inst 1960 25 85 109 14427847

[B49] Raab-Traub N Flynn K The structure of the termini of the Epstein-Barr virus as a marker of clonal cellular proliferation Cell 1986 47 883 889 10.1016/0092-8674(86)90803-2 3022942

[B50] Pitel F Pouzadoux A Langlois P Fillon V Heimel C Douaire M Gellin J Vignal A Mapping of the chicken *SCD1 *locus: assignment of a linkage group to chromosome 6 Animal Genet 1998 29 152 9699283

[B51] Delany ME Robinson CM Goto RM Miller MM Architecture and organization of chicken microchromosome 16: order of the NOR, MHC-Y and MHC-B subregions J Heredity 2009 100 507 514 10.1093/jhered/esp044 19617522

[B52] Ogawa A Solovei I Hutchison N Saitoh Y Ikeda J Macgregor H Mizuno S Molecular characterization and cytological mapping of non-repetitive DNA sequence region from the W chromosome of chicken and its use as a universal probe for sexing Carinatae birds Chromosome Res 1997 5 93 101 10.1023/A:1018461906913 9146912

