# Supplementary figures and images for: Chromosomal integration of an avian oncogenic herpesvirus reveals telomeric preferences and evidence for lymphoma clonality
Source: Herpesviridae. 2010 Dec 7;1:5. doi: 10.1186/2042-4280-1-5 (PMC3063227; doi:10.1186/2042-4280-1-5)

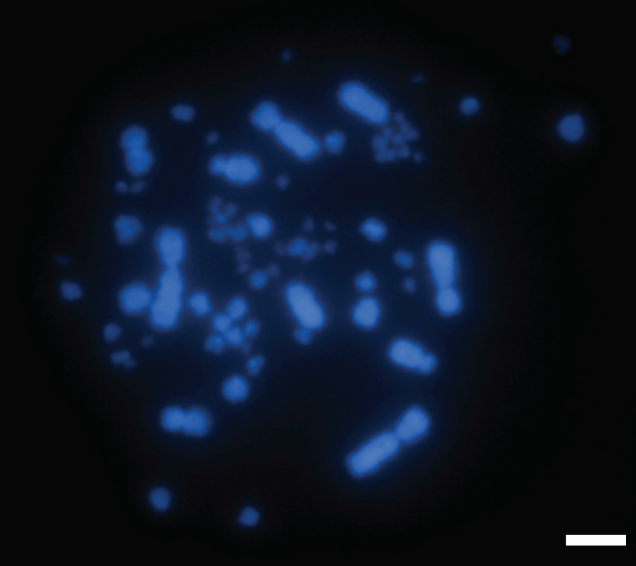

Supplement: Additional file 1 — Hybridization of the MDV BAC probe to uninfected embryonic chicken cells. The MDV BAC was hybridized to chromosomes from uninfected chicken embryo fibroblasts. As shown, the MDV BAC probe did not hybridize to uninfected chicken cells, i.e., there are no FISH signals observed. Scale bar, 5 μm [file 2042-4280-1-5-S1.PDF]

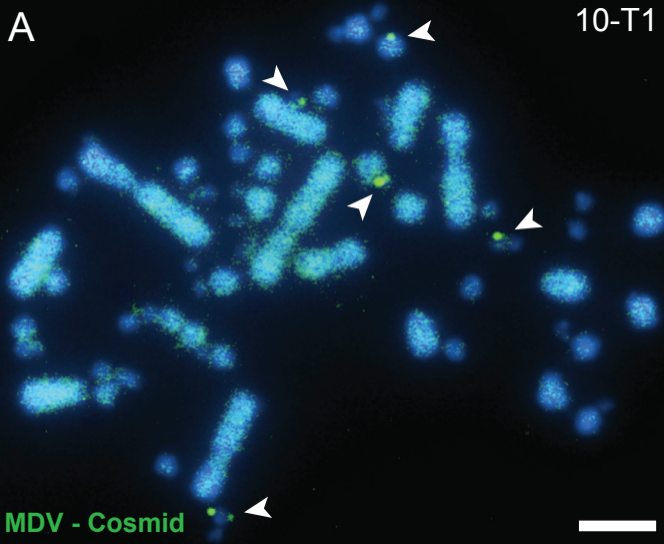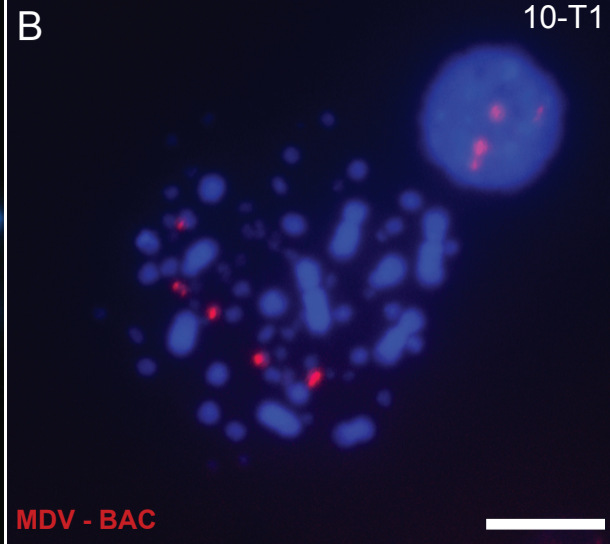

Supplement: Additional file 2 — FISH comparison between two probes: the MDV-BAC and MDV cosmid clones. Panel A displays tumor 10-T1 labeled with the MDV cosmid clones (green) with a total of five integration sites indicated by arrowheads. Panel B displays tumor 10-T1 labeled with the full-genome MDV BAC probe (red) also with a total of five integration sites. Scale bar, 5 μm [file 2042-4280-1-5-S2.PDF]
